# How does long-term odor deprivation affect the olfactory capacity of adult mice?

**DOI:** 10.1186/1744-9081-6-26

**Published:** 2010-05-25

**Authors:** Cathy J Angely, David M Coppola

**Affiliations:** 1 Department of Biology, Randolph-Macon College, Ashland, VA 23005, USA

## Abstract

**Background:**

Unilateral naris occlusion (UNO) has been the most common method of effecting stimulus deprivation in studies of olfactory plasticity. However, despite the large corpus on the effects of this manipulation, dating back to the 19th century, little is known about its behavioral sequela. Here we report the results of standard olfactory habituation and discrimination studies on adult mice that had undergone perinatal UNO followed by adult contralateral olfactory bulbectomy (bulb-x).

**Methods:**

The olfactory performance of UNO mice was compared to matched controls that had unilateral bulb-x but open nares. Both habituation and discrimination (operant) experiments employed a protocol in which after successful dishabituation or discrimination to dilute individual odors (A = 0.01% isoamyl acetate; B = 0.01% ethyl butyrate; each v/v in mineral oil), mice were challenged with a single odor versus a mixture comparison (A vs. A + B). In a series of tests the volume portion of Odor B in the mixture was systematically decreased until dishabituation or discrimination thresholds were reached.

**Results:**

For the habituation experiment, UNOs (n = 10) and controls (n = 9) dishabituated to a 10% mixture of Odor B in Odor A after being habituated to A alone, while both groups failed to show differential responding to a 2% mixture of B in A. However, the UNO group's increased investigation durations for the 2% mixture approached significance (p < 0.06). A replication of this study (7 controls & 8 UNOs) confirmed that controls did not differentiate Odor A and a 2% mixture of B in A but UNOs did not (p < 0.05). For the discrimination experiment, 4 UNOs and 4 controls were shaped to dig in one of two containers of sand that contained the S+ odor (Odor B) to obtain sugar pellet rewards. As in the habituation experiment, UNOs displayed greater olfactory capacity than controls on this task. Controls and UNOs had an average mixture discrimination threshold of 1.6% (± 0.4) and 0.22% (± 0.102) respectively, a difference that was statistically significant (p < 0.02).

**Conclusions:**

Adult mice relying on an olfactory system deprived of odor by naris occlusion from near the time of birth display enhanced olfactory capacity compared to control mice. This counterintuitive result suggests that UNO is neither an absolute method of deprivation nor does it diminish olfactory capabilities. Enhanced olfactory capacity, as observed in the current study, that is a consequence of deprivation, is consistent with recent molecular and physiological evidence that stimulus deprivation triggers compensatory processes throughout the olfactory system.

## Background

Evidence drawn from the visual, auditory and somatosensory systems illustrates the indispensability of ongoing sensory experience for normal anatomical and functional brain maturation [1]. Among the earliest experimental evidence for this form of neural plasticity came from studies of the olfactory system when it was shown that surgically occluding one naris of rabbit pups leads to a subsequent size reduction in the ipsilateral adult olfactory bulb [2]. This effect of UNO, attributed to unilateral sensory deprivation, has been replicated in the rat, mouse, and rabbit among other species [reviewed in [3]]. Indeed, studies of the effects of UNO have produced a large body of empirical evidence consistent with the conclusion that sensory deprivation, beginning early in life, hampers the subsequent development of the olfactory system [reviewed in [3,4]].

In contrast to the numerous studies on the gross anatomical, cellular, and neurochemical sequelae of UNO in the mucosa and olfactory bulb, few studies have addressed, using behavioural tests, the functional capacity that is retained. However, a study that used air-dilution olfactometry and operant conditioning methods, was able to show that adult rats receiving UNO perinatally and adult contralateral bulb-x could detect and discriminate very dilute odors near their detection levels prior to surgery [5]. This result was rather surprising given the reported 25% reduction in size of the ipsilateral bulb in adult rats following perinatal UNO [6]. In a more naturalistic, if less taxing, test of olfaction, 10 day old mice that had undergone UNO and contralateral bulb-x the day after birth could still use maternal pheromone to find their mother's nipple [7]. Collectively, these results suggest that UNO does not create absolute deprivation nor does it lead to marked anosmia in the ipsilateral olfactory system. Nevertheless, the extent of behavioral deficit in adults, caused by perinatal UNO, remains unknown. In this study two commonly used behavioral tests of olfaction: habitutation-dishabituation, and an operant conditioning paradigm were used to compare odor generalization and discrimination in mice that received either perinatal UNO and adult contralateral bulb-x or only contralateral bulb-x (controls). The results support the counterintuitive conclusion that the olfactory capacity of the mice in the UNO group exceeded that of controls.

## Methods

Mice used in this study were males and females from the CD-1 strain born in the Randolph Macon Animal facility to timed-pregnant dams obtained from Charles River Labs, (Wilmington, MA, USA). All animal procedures adhered to the NIH Guide to the Care and Use of Laboratory Animals and were approved by an Institutional Animal Care and Use Committee.

### Naris Occlusion

On the day after birth mouse pups were induced with saturated isoflurane vapor and anesthetized by hypothermia. Subsequently pups received either UNO (right or left) by cautery as previously described [8] or a sham procedure involving a small cautery application to the muzzle positioned so as not to restrict airflow to either naris. Once the UNO or sham surgeries were complete, pups were placed near an incandescent lamp until they became ambulatory after which they were returned to their litter. Following surgery 4% lidocaine was applied daily to the cautery wound until healing was complete. Mice whose occluded naris remained patent 48 hrs after surgery, as judged by visual inspection under 20 × magnification using a soap solution to detect air movement, were excluded from the study. While an exact tally of successful occlusions is not made, the success rate in our lab is estimated to be in the 70-80% range.

After initial behavioral testing the occluded naris in one mouse was surgically reopened under anesthesia (ketamine/zylazine, 90/9/mg/kg IP) by perforating the scar tissue at the site of occlusion with a #7 insect pin. In order to maintain the patency of the reopened naris a 2 mm length of 22 ga cardiac cannula was fitted in the orifice and held in place with cyanoacrylate cement. Patency of the reopened naris was established by recording the output of a thermistor (ADInstruments, Colorado Springs, CO, USA) placed ~2 mm inside the new orifice while the animal was still under anesthesia (see results).

### Olfactory Bulbectomy

To test the olfactory capabilities of mice forced to use only their olfactory system components ipsilateral to UNO, the olfactory bulb was removed on the side contralateral to the occluded naris [5].

Unilateral bulb-x was performed by first anesthetizing the subjects with a combination of ketamine, xylazine, and butorphenol (90/9/5 mg/kg IP). When deep anesthesia was achieved, determined by the loss of withdraw reflex, the surgery proceeded. A ~1 cm incision was made in the skin overlying the suture of the caudal and rostral bones on the side of the head contralateral to naris occlusion. After the skin was reflected a ~1 mm dia craniotomy was made in the nasal bone overlying the olfactory bulb with a dental drill. Under visual guidance, aided by a stereosurgical microscope, bulbar tissue was aspirated unilaterally through the craniotomy with attention to removal of the portion of the bulb that lies under the frontal pole. The cavity created by removal of the bulb was then packed with gel foam. Subsequently, the craniotomy was sealed with bone wax and the incised skin was sutured closed and treated with topical antibiotic ointment containing lidocaine. Afterwards, mice were placed under an incandescent lamp until they awakened. Starting the day after surgery the condition of each bulb-x mouse was monitored daily until recovery.

For control animals with intact nares, the bulb to be removed---right or left---was chosen by coin toss.

### Odorants

The odorants used were the purest commercially available and were diluted daily from pure stores kept at -4°C. Iso-amyl acetate (IA), obtained from Aldrich (Milwaukee, WI, USA) at 97+% purity, and ethyl butyrate (EB), obtained from Fluka (Milwaukee, WI) at 99.7% purity, were diluted in mineral oil v/v to a working stock concentration of 0.01%. All subsequent uses of the term "stock" in this paper refer to a 0.01% v/v dilution of odorant in mineral oil. The stimulus set consisted of further dilutions of EB stock in IA stock as follows: 10%, 2%, 0.4%, 0.08%, 0.016% and 0.0032% EB. These percentages represent the volume portion of 0.01% EB stock in 0.01% IA stock. Thus, a 10% dilution refers to one part 0.01% EB in nine parts 0.01% IA.

### Odor Habituation-Dishabituation

Initially, nine control mice that had received unilateral bulb-x as adults and ten UNO mice that had received contralateral bulb-x as adults were tested. Following the testing of these mice an additional cohort was tested, four months older at the time of testing than the first. This group consisted of seven mice that received only unilateral bulb-x and eight mice that received UNO and contralateral bulb-x.

The treatment conditions of the mice in this study were unknown to the behavioural observer in order to minimize the possibility of investigator bias.

Twenty-four hours before testing, a 2.5 cm dia stainless steel tea strainer was hung from the home-cage cover of each mouse to be tested. The tea strainer contained one round, 2.5 mm dia, piece of filter paper with 40 μl of mineral oil adsorbed onto it. Testing began after each mouse, still in the presence of the tea strainer affixed to the home cage, was individually acclimated for 30 min to a test room apart from the vivarium.

Powder free Neoprene gloves were worn during stimulus changes to avoid contaminating the habituating stimulus with new odors.

To begin a test the filter paper containing mineral oil present during the acclimation period was removed and replaced with new filter paper that had 40 μl of IA stock adsorbed onto it. The duration of investigation, defined as 'snout judged to be within 1 cm of the tea strainer,' was recorded using a stopwatch during a 50 sec trial. In practice the vast majority of stimulus investigation was unambiguous with the mouse actively sniffing with snout within a couple of mm of the tea strainer. Once the trial ended the cage top with the tea strainer was removed and a clean top was placed on the cage. During the three min intertrial interval new filter paper with fresh IA stock odor replaced the paper used in the previous trial. Then the tea strainer with fresh IA odor was replaced on the cage to start the next trial. These procedures were repeated for six habituation trials with IA odor. Before the seventh or "test" trial, the filter paper was removed during the intertrial interval and replaced with new filter paper upon which 40 μl of EB stock was adsorbed. As before, the investigation duration within a 50 sec interval was recorded. The experiment was repeated daily using decreasing dilutions of EB stock in IA stock as the test odorant.

Testing ended when the group of mice failed to show statistically significant (p < 0.05) dishabituation (i.e. increased investigation times) to the EB-IA mixture. To establish dishabituation a paired t-test was used to compare investigation times in the last (6^th^) habituation trial to the investigation times in the following test (dishabituation) trial. A paired t-test to compare the first and last (6^th^) habituation trials was used to establish that habituation had occurred. Also, ANOVA was used to test for the linear trends of the habituation trials (GraphPad, La Jolla, CA, USA). Statistical results confirming habituation were deemed sufficiently tangential to omit from this report in the interest of brevity.

### Odor Discrimination

Four control mice with unilateral bulb-x and four UNO mice with contralateral bulb-x were tested in this part of the study. Mice were placed on food restriction to maintain their weight between 80-85% of their free-feeding level. Shaping and testing trials were performed in a 47 cm × 25.4 cm × 20 cm polypropylene cage. Within the cage there were two, 10 cm dia by 1.8 cm deep, circular dishes. Each dish contained half of a three cm circle of filter paper with five μl of odorant stock adsorbed onto it. Filter paper was affixed to the side of the dishes so as to protrude above the top edge using double-sided tape. The dishes were filled with sand and placed on opposite sides of the test cage.

During the initial shaping trials one dish contained filter paper with five μl of mineral oil adsorbed onto it, while the other dish contained filter paper with five μl of EB stock odor. The dish with EB odor (S+) held a 45 mg sucrose reward (TestDiet, Purina Mills, Richmond, ID, USA). The reward was initially placed on top of the sand in front of the filter paper and was odorized by the placement of one μl of EB stock on the outer surface of the sugar pellet. The mouse began the trial in a 20 cm dia cylindrical start chamber within the test cage. This chamber was removed to begin the test and a Plexiglas top was placed over the cage to allow observation. Once a mouse consistently retrieved and consumed the reward during trial periods, the reward was incrementally buried deeper into the sand in subsequent shaping trials until the mouse would dig vigorously to find the reward completely buried by sand.

In the next phase of shaping the mouse was required to find the reward using only the EB odorant adsorbed to the filter paper on the dish wall with no odor added to the sugar pellet. In these shaping sessions the filter paper in one dish contained the EB stock (S+) and the other dish contained filter paper with IA stock (S-). If the mouse dug in the dish containing the S-odor, it was removed and placed into its home cage for 20 sec after which it was allowed to start another trial. Each time the reward was found and consumed the mouse was placed back in the start chamber while the next shaping trial was set. Once a mouse consistently went to the dish containing the S+ odorant it was considered to be ready for testing.

Testing consisted of a block of six one-min trials in which the amount of time spent investigating the sand directly in front of the filter paper and digging in each dish were recorded with a stopwatch. Dishes remained on opposite ends of the cage and odor concentrations were identical to those used during shaping. A coin toss determined the end of the test cage where the dish containing the S+ odor and the S- odor would be positioned for each trial. During test trials no reward was available as a precaution against cueing on odors from the sugar pellet itself. During the intertrial interval mice were placed in the start chamber. Either one or two (decided by coin toss) reinforcement trials with sugar rewards were interspersed between test trials to prevent extinction of the shaped behavior. These reinforcement trials were not used in data analysis.

After reinforcement and test trials were completed, total investigation times in the S+ and S- dish were compared for the test trials using the Mann-Whitney test. This test was chosen because there was no significant correlation of S+ and S- investigation times within a given observation period thus making paired comparisons inappropriate and because it tends to be conservative. In any event, using other logical alternative statistical methods (t-test and/or paired comparisons) made little difference in our overall conclusions (data not shown).

Following a block of trials for which there was significantly (p < 0.05) more investigation of S+ versus S-, S+ was changed to a greater dilution of EB stock in IA stock for the next test. If there was not a significant difference in investigation times for a given S+ and S- pair, then a mouse was retested at the same S+ concentration on the following day. If a mouse again failed to show a significant difference in investigation duration between the S+ and S- it was considered to have reached its threshold of discrimination. On this point we acknowledge departing from the more common practice in psychophysics of setting an arbitrary (nonstatistical) discrimination criterion, which is likely a more conservative approach.

Between-group statistics were of secondary consideration in this part of the study because it was impractical to hand shape the behavior of a large group of mice. It was deemed to be more instructive to exhaustively test a small number of mice than to superficially test a larger group for the purposes of doing between-group statistics. The primary interest was not to make quantitative as much as qualitative comparisons between UNO and control mice. Given the lack of prior work on this topic it was considered more important to determine if *any *UNO mice could perform near the level of *any *control mice than it was to know how the two groups compare quantitatively.

### Histology

After behavioral testing was completed mice, except those noted below, were prepared for histological verification of bulb-x. First, mice were given a deeply anesthetising dose of Euthasol (70 mg/kg; Butler, Dublin, OH, USA), and transcardially perfused with phosphate buffered saline followed by 4% paraformadahyde. Heads were then removed, trimmed of excess tissue, post-fixed for 24 hours and decalcified in RDO (Apex Engineering Products; Aurora, IL, USA). Subsequently, heads were placed in 30% sucrose for 24 hrs, frozen in isopentane cooled with dry ice and cryostat sectioned in the horizontal plane at 40 μm. Sections were then mounted on Superfrost slides (Brain Research, Waltham, MA, USA) and stained for Nissl substance.

To further investigate the connectivity of olfactory receptor neurons on the bulb-x side, one of the UNO mice used in the discrimination tests had 5 μl of 20% HRP infused into the open naris. After a 48 hr survival time the mouse was processed according to the procedure described by Hunt and Slotnick [5] to determine if any olfactory receptor cell terminals could be detected in remnants of the aspirated olfactory bulb. The cell internalized HRP was made visible with a TMB substrate kit (Vector Labs, Burlingame, CA, USA) after which the sections were counterstained with Neutral Red (Vector Labs).

## Results

### Habituation-Dishabituation

In a series of preliminary tests on 13 untreated adult mice (data not shown) it was determined that: (1) the CD-1 strain of mice used in this study did not have an inherent preference for investigating either of the tests odors, (2) that either IA stock or EB could serve as habituation or dishabitutation odors, and (3) that normal mice did not show statistically significant dishabituation to a 2% mixture of EB stock in IA stock after being habituated on IA, though they did show dishabituation to a 10% mixture. These data were the basis for the dilution series selected for testing the experimentally manipulated mice described below.

**Figure 1:**
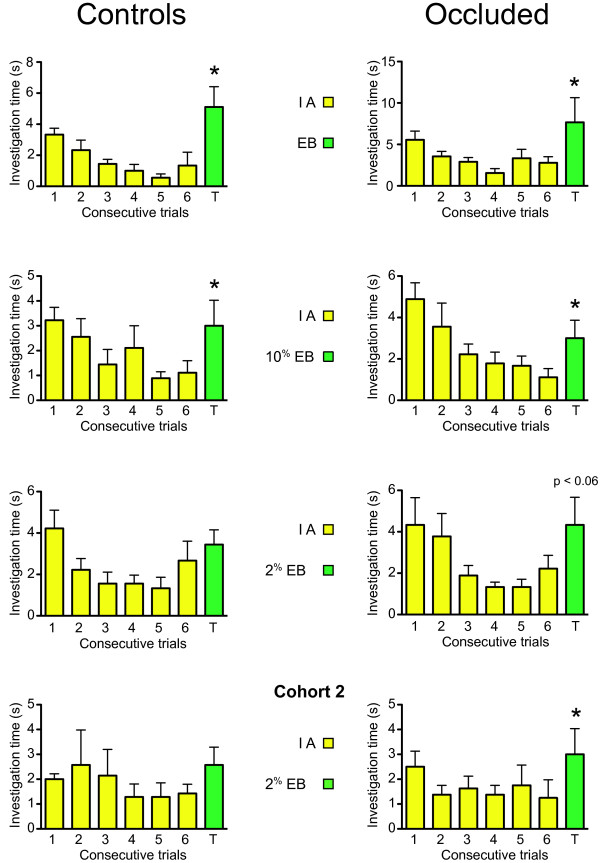
**Habituation Results**. Mean (± SEM) investigation times for unilateral bulb-x mice during 50 sec habitation (1-6) and dishabituation (T) trials. Data in the left column (controls) are from adult mice that had normal nares (n = 9). Data in the right column (occluded) are from adult mice that received perinatal UNO contralateral to their subsequent adult bulb-x (n = 9). Note that results from the same groups of mice, retested with increasingly diluted test odors, are shown in the first three rows. The fourth row depicts data from a replication of the experiment with a novel cohort of mice (controls n = 7; occluded n = 8). However, only data for the most diluted test odor (2% EB stock in IA stock) is shown. Asterisks denote statistical significance of differences in mean investigation times toward the novel stimulus ("T" = trial seven) compared to habitation stimulus in trial six (paired t-test); * p < 0.05

The habituation-dishabituation results for control mice and UNO mice are shown in Fig. 1. In tests using single odor stock solutions both controls (n = 9) and occluded mice (n = 9) showed significant dishabituation (t = 2.27, p < 0.03 and t = 2.03, p < 0.04, respectively). This was also true when the novel odor was 10% EB in IA stock (t = 1.85, p < 0.05 and t = 2.16, p < 0.03, respectively). However, when the novel odor stock solution was further diluted to 2% in IA stock, both groups failed to show dishabituation, (for controls: t = 1.14, p < 0.14) though results from the UNO group approached the significance level (for UNO group: t = 1.75, p < 0.06). To confirm and extend these results a second cohort of mice was tested. The mice in this group were four months older than the first cohort but otherwise were treated identically. Only data from the responses to the 2% dilution of EB stock in IA stock are shown in Fig. 1. Responses to the other dilutions were statistically similar in habituation and dishabituation to the first cohort and were thus excluded from the data presentation. Results from the second cohort confirmed those from the first cohort that controls investigated the 2% dilution of EB stock in IA stock (t = 1.29, p > 0.12, n = 7) as if it were pure IA stock. However, as suggested by the nearly significant t-value from the first cohort, occluded mice in the second cohort evidenced significantly greater interest in the 2% EB solution compared to the last presentation of pure IA stock (t = 2.70, p < 0.02, n = 8).

### Odor Discrimination

Results shown for this part of the study are derived from repeatedly testing four control mice and four UNO mice. Odor discrimination threshold for an individual mouse was defined as the mixture concentration (S+ = EB stock v/v in IA stock) it failed to discriminate from the single component stimulus (S- = IA stock) on two consecutive days as determined by non-significant Mann-Whitney tests of investigation times. For simplicity of presentation the numerous individual critical values and probabilities levels for the statistical tests have been omitted for the individual stimulus pairs.

All four of the control mice discriminated the pure IA stock solution from a mixture of EB stock in IA stock at dilutions greater than those discriminated by the control mice in the habituation tests. Thus, both cohorts of control mice in the habituation study generalized pure IA stock solution and a 2% mixture of EB stock in IA stock, while all four control mice tested with reinforcement discriminated these stimuli. However, only one control mouse (data not shown) could discriminate IA stock from higher dilutions than 2% EB stock in IA stock solution. This mouse discriminated pure IA stock from 0.4% EB stock in IA stock but failed at the 0.08% dilution. Unfortunately, this mouse was inadvertently not tested a second day with the 0.08% mixture so it is unknown if it could have discriminated at lower mixture concentrations based on our threshold criterion (see methods).

**Figure 2:**
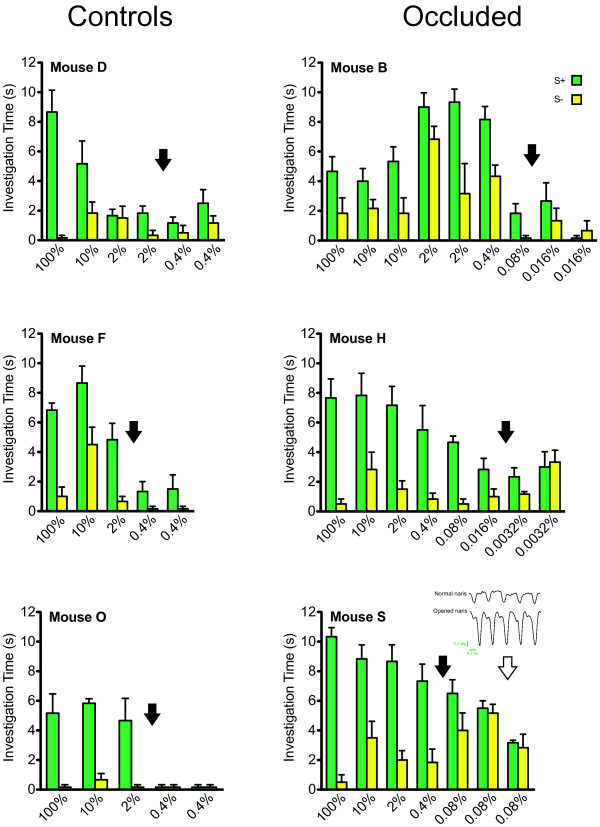
**Discrimination Results**. Mean (± SEM) digging times for unilaterally bulb-x mice during 60 sec discrimination trials comparing two sand-filled dishes containing either S+ or S- odors. Data in the left column (controls) are from three of four trained adult mice that had normal nares. Data in the right column (occluded) are from three of four trained adult mice that had a naris occluded on the first postnatal day positioned contralateral to their subsequent adult bulb-x. Mice were tested on their ability to discriminate the S- and increasingly diluted S+ in S- stock (see methods). Filled arrows designate threshold as determined by lowest dilution (just left of arrow) at which the mouse showed significant discrimination by Mann-Whitney test (p < 0.05) in a six trial block. Note that a mouse had to fail to discriminate a given dilution on two consecutive days to establish threshold. In the case of *Mouse S *(lower right panel), after threshold was determined its formerly occluded naris was opened surgically (open arrow). The traces in the inset are recordings from a thermistor probe placed just inside the external nares after naris opening. These traces establish that respiration was restored through the surgically reopened naris. After a two-day recovery period following naris reopening, *Mouse S *again failed to discriminate the dilution (0.08% S+ stock in S- stock) it had previously failed on during threshold testing.

In contrast to the control mice but consistent with the habituation results, UNO mice tended to outperform control mice on the discrimination tests (Fig. 2). Two of the four UNO mice achieved thresholds at least half a log unit lower than the best performing control mouse and the other two control mice had the same threshold as the best performing control mouse. Put differently, of the eight mice tested, the three with the highest thresholds were from the control group and the three with the lowest thresholds were from the UNO group. Notably, Fig. 2 does not display the data from one of the UNO mice because it died of unknown causes before histological confirmation of bulb-x could be done.

One of the UNO mice (Mouse H; see Fig. 2) achieved the feat of discriminating pure IA stock from a 0.016% mixture of EB stock in IA stock. The lawful monotonic decline in S+ investigation-times with increasing dilution of the stimulus provides further evidence that odor and not some other cue was the basis of this animal's discriminations.

In another UNO mouse (Mouse S; see Fig. 2) the closed naris was surgically reopened after which retesting occurred at the previous subthreshold mixture concentration. However, this subject's ability to discriminate was not improved by reopening the occluded naris.

While, as noted in the methods, group comparisons were not the focus of this part of the study, the control group and UNO group had an average mixture discrimination threshold of 1.6% (± 0.40) and 0.22% (+ 0.10) respectively, a difference that was statistically significant (p < 0.02, t-test) despite the small sample size.

### Histology

**Figure 3:**
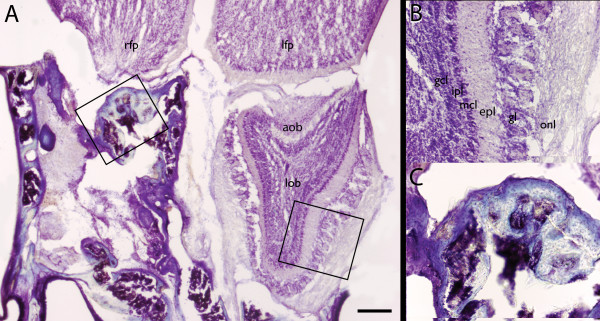
**Histological Confirmation of Bulb-x**. Photomicrographs of Nissl-stained horizontal section (rostral toward bottom) through cortical frontal pole and olfactory bulbs of unilaterally bulbectomized mouse (*Mouse H *in Fig. 2). **A**. Lower magnification view: Note scar tissues and lack of organized neuropil where right olfactory bulb was removed (left side). **B**. Higher magnification view from intact left bulb ipsilateral to naris occlusion showing bulbar lamina (see region of interest in **A**, right). Note abnormally thin lamina relative to typical adult mouse olfactory bulb histology. **C**. Higher magnification view from right side ipsilateral to bulbectomy (see region of interest in **A**, left). Note apparent lack of organized neuropil, particularly any detectable glomerular structure. This disorganization was confirmed in serial sections throughout the extent of the olfactory bulb in naris occluded subjects and in a sample of normal mice. *aob *= accessory olfactory bulb; *epl *= external plexiform layer; *gcl *= granule cell layer; *gl *= glomerular layer; *ipl *= internal plexiform layer; *lfp *= left frontal pole; *lob *= left olfactory bulb; *mcl *= mitral cell layer; *onl *= olfactory nerve layer; *rfp *= right frontal pole; Scale bar in **A **= 250 μm.

Bulb-x mice from both the habituation study and discrimination study whose brains were examined histological, had greater than >95% of the targeted olfactory bulb ablated as subjectively judged from the Nissl stained serial sections. However, as noted previously, one mouse in the discrimination study died before histology could be performed (Fig. 3). Importantly, several mice in the UNO groups had no detectable remaining bulb tissue on the ablated side. Since the amount of remnant bulbar tissue did not predict behavioral performance none of the mice were excluded from the study. This was also the justification for foregoing a detailed quantitative reconstruction of postmortem histology for the purpose of determining exact percentages of bulb remnants.

**Figure 4:**
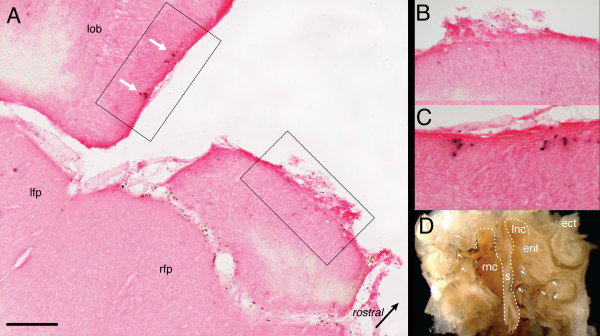
**HRP Labelling of Bulb and Mucosa**. **A-C**. Photomicrographs of HRP labelling (TMB substrate) and Fast Red counterstaining in a horizontal section through cortical frontal pole and olfactory bulbs of unilaterally bulbectomized mouse (*Mouse S *in Fig. 2). **A**. Arrows mark HRP foci in glomerular layer of preserved bulb. **B**. Higher magnification view on side ipsilateral to bulbectomy showing bulb stump rostral to cortical frontal pole (see region of interest in **A**, right). Note lack of HRP positive axonal ingrowth to bulb vestige. **C**. Higher magnification view of mediocaudal region of preserved bulb (see region of interest in **A**, left). Note HRP positive axonal ingrowth to glomerular region. **D**. Coronal view of fixed but undecalcified portion (see ragged borders) of caudal nasal cavity with HRP labelling. HRP had been infused into right naris 48 hrs prior to labelling. Note HRP in presumptive olfactory receptor neurons of both nasal fossa (arrows) but label is limited on the left side to ventral turbinates. *ect *= ectoturbinate; *ent *= endoturbinate; *lfp *= left frontal pole; *lnc *= left nasal cavity; *lob *= left olfactory bulb; *rnc *= right nasal cavity; *rfp *= right frontal pole; *s *= nasal septum; Scale bar in **A **= 250 μm.

To confirm further the lack of connectivity between the patent nasal cavity and ablated bulb in a UNO mouse, HRP was injected in the still open naris of Mouse S (see Fig. 2) after its behavioral testing was completed [5,9]. As noted previously, this subject performed better than three of the four control mice (Fig. 2). After a 48-hour survival period HRP reaction product was found in the cells of the contralateral as well as the ipsilateral olfactory mucosa (Fig. 4D). Presumably HRP injected into the open naris gains access to the occluded nasal cavity by flow through the nasopharyngeal canal or by retronasal reflux [5]. Despite the extensive labelling of presumptive olfactory receptor neurons, no axonally transported HRP label was found in the remnant tissue of the bulb targeted for ablation nor was any found in anterior forebrain (Fig. 4B). However, axonally transported reaction product was found in glomeruli of the intact bulb (Fig. 4C).

## Discussion

Odor habituation has been used in studies of odor generalization and has been touted to be the preferred method, compared to operant conditioning, for studying the output of certain kinds of neural processing such as those that underlie olfactory coding [10,11]. In the current study habituation tests were chosen for their simplicity. The expectation was that UNO animals, lacking a normal path of odor access from the environment through an external naris to an intact central olfactory system, would have marked odor deficits compared to control mice. However, this prediction proved to be incorrect. In the first cohort of mice the UNO group had the same threshold of mixture discrimination as the control group. Indeed, for the lowest mixture concentration tested, 2% EB stock solution in IA stock solution, data for the UNO group approached statistical significance for discrimination. This intriguing result prompted the testing of a second cohort of UNO and control mice confirming that UNOs dishabituate to a 2% mixture of EB stock in IA stock while controls did not differentiate this mixture and IA stock alone.

One potential explanation for this surprising result is that the UNO group had spared olfactory bulb tissue on the non-occluded side. However, this possibility was not supported by histological evidence. Another possibility is that some non-olfactory cue supported habituation-dishabituation behavior. However, mice could not see or contact the stimuli and therefore other sensory systems, including other chemosensory ones, are unlikely to have been the basis of behavioural responses.

To further investigate these results a smaller group of UNO and control mice were shaped to discriminate odor cues using operant methods in a standard digging paradigm [e.g. [12]]. It is known that habituation-dishabituation tests do not provide measurements of absolute thresholds [10]. For example, rats generalize certain odor pairs in a habituation test that they can learn to discriminate under reinforcement [10]. This fact was born out in the current operant study by the lower thresholds for mixture discrimination by both UNOs and controls amounting to at least half a log-unit in magnitude compared to the habituation tests (Fig. 2). But here too the UNO group outperformed the control group in ability to discriminate the S+ mixture from the S- single odor.

Since UNO mice could make fine odor discriminations, we next asked whether opening the occluded naris could improve olfactory performance. Unfortunately, due to the unexpected difficulty of this seemingly simple surgery only one mouse was tested after naris reopening. Though airflow was restored through the reopened naris as established by a thermistor probe, discrimination threshold was unchanged.

As was the case for the habituation experiment, histological examination of the mice used in this part of the study confirmed total or near total loss of organized bulbar tissue on the side targeted for ablation. Moreover, anterograde transport of HRP failed to establish any functional bulbar connections on the side of bulb-x in an animal with discrimination performance better than most controls (Fig. 4). Thus, it is unlikely that the better-than-normal performance of UNO mice in the discrimination tests can be explained by incomplete bulb-x.

Taken together the results of the current study were quite unexpected. Our initial interest was to test the olfactory capabilities of an olfactory system raised under the UNO regime after reopening the naris to allow normal access of odors to the nasal cavity. The testing of the still occluded animals---the bulk of the data reported here---was originally planned as a control condition to be compared to animals after naris reopening. However, our results establish a striking preservation of olfactory capabilities in UNO animals. Moreover there was no improvement in olfactory discrimination threshold after naris reopening, though clearly this result requires further replication.

It is unclear how to reconcile the preservation of olfactory capabilities in UNO mice observed in this study with the legion seemingly negative effects that have been reported for this form of deprivation [reviewed by [3,4]]. For example, it is well established that perinatal UNO leads to the development of an ipsilateral olfactory bulb that is ~25% smaller than a normal bulb [e.g. [2,13,14]] with nearly every bulbar layer effected [15]. Cell labelling studies have established that the effect of UNO on bulb size is predominantly due to decreased cell survival rather than a decrease in neurogenesis [[16,17]]. A large body of additional anatomical and physiological evidence points to the detrimental effects of UNO on the developing olfactory system [reviewed in [3,4]]. Given this corpus, it was reasonable to expect that UNO mice with contralateral bulb-x would have diminished olfactory capabilities, in part because of the blockade of the odor path. Further it could be predicted that UNO mice with a reopened naris would still have reduced capabilities because of the many reported detrimental effects of perinatal UNO to the developing ipsilateral bulb. In contrast to these findings, Hunt and Slotnick [5] were unable to show a marked effect, in an operant task, of perinatal UNO combined with adult contralateral bulb-x on the ability of rats to detect and discriminate fairly low concentrations of odor vapor. They demonstrated only a small decline in task acquisition times in UNO rats compared before and after bulb-x but unfortunately their study lacked control subjects without naris occlusion and they made no attempt to determine psychophysical thresholds. Nevertheless, these findings established that UNO does not prevent odor vapors from reaching the sequestered nasal cavity, a fact that the authors attributed to the existence of a nasopharyngeal canal that connects the two nasal vaults and/or to a potential retronasal odor path [5].

The results of the current study in mouse confirm and extend those of Slotnick and colleagues [5,18]. However, in contrast to their work, UNO mice in the current study actually outperformed control mice on a habituation task and a discrimination task. How can this counterintuitive result be accounted for? One possibility is suggested by several recent studies demonstrating that many changes in the olfactory system following UNO appear to be 'compensatory' in that they are in the direction of preserving olfactory function in the face of stimulus deprivation [3,19].

For example, Tyler and colleagues [20], using whole-cell voltage-clamp, showed that two weeks of UNO in rats, beginning on the second postnatal day, increases the probability and quantal content of neurotransmitter release at ipsilateral primary olfactory synapses in the bulb. Explaining this physiological effect, these authors found that vesicular glutamate transporter and glutamate receptor subunits, key functional synaptic components, were up-regulated in the ipsilateral bulb. In addition voltage-clamp recordings of spontaneous and olfactory-nerve-evoked activity in the predominant second-order neurons of the bulb, including mitral cells, demonstrated that UNO strengthens synapses in down-stream components of the olfactory circuit [20].

As noted above, increased cell death occurs in the ipsilateral bulb following perinatal UNO that is particularly prevalent among granule cells and other inhibitory neurons of the bulb [16]. However, the depletion of ipsilateral granule cells following UNO appears to be compensated for by an increased excitability among the remnant granule cell population [17].

These apparent compensatory processes may explain why electrophysiological studies have failed, for the most part, to show significant differences in the overall circuit properties of the ipsilateral bulb or its central targets following perinatal UNO [e.g. [21]].

Compensatory responses to UNO also have been reported recently in the olfactory periphery. For example, components of the sensory transduction cascade, including modulatory elements, differ in abundance in ipsilateral olfactory sensory neurons (OSNs) compared to contralateral or control OSNs in a manner consistent with compensation [[8,22]]. These observations based on Western blotting and semi-quantitative analysis of immunolabeled OSN or olfactory mucosal tissue have recently been confirmed and extended at the RNA level using microarrays [23]. Presumably, these responses to UNO at the molecular level underlie the enhancement recently reported in electro-olfactogram amplitudes from the ipsilateral versus contralatateral or normal olfactory mucosa [24]. This latter result implies that more OSNs are recruited on the occluded side, compared to controls, by a given odor or those that respond do so with a greater amplitude [3].

## Limitations

The most serious limitation of this study was the small sample of subjects used in the discrimination experiment. While results for subjects within the two comparison groups were consistent and statistically significant, such small sample sizes are a matter of concern. The difficulty of using operant methods with mice together with the need for complete postmortem histological verification of bulb-x is a serious impediment to doing parametric studies focusing on group differences. These complications may explain why only one previous study addressed the questions that were the focus of this report [5].

Another limitation of this study relates to the assumption that olfactory receptor neurons ipsilateral to bulb-x do not regain functional connections to the brain. Our HRP experiment provides some assurance that these neurons do no make connections with bulb remnants or forebrain on the ipsilateral side but leave open the possibility that they project new axons to the contralateral side following bulb-x. We can find no evidence in the literature that this actually occurs but it remains an untested assumption. Of course, even if such decussation occurred it would not explain why UNO mice, on average, outperformed controls in our behavioural studies.

## Conclusions

Given the growing body of evidence in support of compensatory responses to odor deprivation, our provisional conclusion is that the superior behavioural performance of UNO mice in this study is the combined result of these various mechanisms. From an evolutionary perspective such compensatory processes, which appear to be implemented at various levels of the olfactory system from sensory neuron to behavior, would seem highly adaptive. Given any sensory system's finite dynamic range, nature may have built in sufficient plasticity to continuously adjust responses to maximize the useful information transferred about the environment [25]. This is why sensory systems modulate their output to report *changes *in the environment rather than static levels of the stimulus [see [26] for an example in vision]. Sensory adaptation is a short-term example of this mechanism that has been studied extensively, both empirically and theoretically, in many sensory systems [e.g. [27]]. The effects of long-term deprivation on the olfactory system, such as that seen following perinatal UNO, can be understood similarly, though cellular mechanisms, time course, and reversibility may be quite different than those seen in adaptation. From this viewpoint, animals exposed to 'noisy' or 'enriched' odor environments might be expected to show changes opposite to those reported here for the deprived state. Interestingly, just this kind of push-pull arrangement has been reported in levels of certain modulators of the transductory cascade in OSNs that were exposed to enriched versus deprived odor environments [8,22]. Also, odor rich environments have opposite effects to odor deprivation in the bulb, leading, for example, to enhanced granule cell survival [28].

## Competing interests

The authors declare that they have no competing interests.

## Authors' contributions

CJA: Collected behavioral and some anatomical data. Planned some parts of experiments.

DMC: Collected some anatomical data, planned some experiments, did statistical analysis, made graphs, and wrote manuscript.

Both authors read and approved the final version of the manuscript.
